# In Ovo Vaccination with Recombinant Herpes Virus of the Turkey-Laryngotracheitis Vaccine Adjuvanted with CpG-Oligonucleotide Provides Protection against a Viral Challenge in Broiler Chickens

**DOI:** 10.3390/v15102103

**Published:** 2023-10-17

**Authors:** Carissa Gaghan, Matthew Browning, Abdelhamid M. Fares, Mohamed Faizal Abdul-Careem, Isabel M. Gimeno, Raveendra R. Kulkarni

**Affiliations:** 1 Department of Population Health and Pathobiology, College of Veterinary Medicine, North Carolina State University, Raleigh, NC 27607, USAamfares@ncsu.edu (A.M.F.); 2 Health Research Innovation Center 2C58, Faculty of Veterinary Medicine, University of Calgary, Calgary, AB T2N 1N4, Canada; mfabdulc@ucalgary.ca

**Keywords:** infectious laryngotracheitis, vaccine, protection, CpG oligonucleotide, adjuvant, chickens

## Abstract

Infectious laryngotracheitis (ILT) is an economically important disease in chickens. We previously showed that an in ovo adjuvantation of recombinant herpesvirus of the turkey-Laryngotracheitis (rHVT-LT) vaccine with CpG-oligonucleotides (ODN) can boost vaccine-induced responses in one-day-old broiler chickens. Here, we evaluated the protective efficacy of in ovo administered rHVT-LT + CpG-ODN vaccination against a wild-type ILT virus (ILTV) challenge at 28 days of age and assessed splenic immune gene expression as well as cellular responses. A chicken-embryo-origin (CEO)-ILT vaccine administered in water at 14 days of age was also used as a comparative control for the protection assessment. The results showed that the rHVT-LT + CpG-ODN or the CEO vaccinations provided significant protection against the ILTV challenge and that the level of protection induced by both the vaccines was statistically similar. The protected birds had a significantly upregulated expression of interferon (IFN)γ or interleukin (IL)-12 cytokine genes. Furthermore, the chickens vaccinated with the rHVT-LT + CpG-ODN or CEO vaccine had a significantly higher frequency of γδ T cells and activated CD4+ or CD8+ T cells, compared to the unvaccinated-ILTV challenge control. Collectively, our findings suggest that CpG-ODN can be used as an effective adjuvant for rHVT-LT in ovo vaccination to induce protective immunity against ILT in broiler chickens.

## 1. Introduction

Infectious Laryngotracheitis (ILT), caused by an alpha-herpes virus, is a highly contagious respiratory infection in chickens. The ILT virus (ILTV) can rapidly spread across broiler houses, resulting in increased rates of morbidity and mortality, decreased bird performance, and significant economic losses to poultry producers [1]. Currently, there are two types of vaccines available for the control of this disease, live-attenuated or modified vaccines such as the chicken-embryo origin (CEO) or tissue-culture-origin (TCO) vaccines, and recombinant ILT (rILT) vaccines that include the herpesvirus of turkeys (rHVT-LT) or fowl poxvirus (rFPV-LT) vaccines [2,3]. Live-attenuated vaccines, specifically the CEO vaccine, which is typically given via eye-drop or in water, can effectively prevent the clinical disease and mortalities associated with ILT. However, the major drawback of the CEO vaccination is that it causes an increased virulence of the virus through bird-to-bird transmission [4,5]. Additionally, the use of this vaccine in broiler flocks can result in spillover of the virus to other broiler flocks, causing transmission of the disease with subsequent infection, morbidity, and mortality [6,7]. On the other hand, rILT vaccines, which are approved for in ovo vaccination, have been shown to reduce the severity of clinical disease and help to improve bird performance [3]. We previously observed that the in ovo vaccination of rHVT-LT vaccination resulted in a significant reduction in tracheal ILTV replication, reduced clinical signs, and increased body weights in broilers [8,9]. Despite these benefits, several reports have indicated that the rHVT-LT vaccine is not as effective as the CEO vaccine, primarily due to poor immunogenicity leading to suboptimal protective immunity in birds post-viral challenge [3,9,10,11].

Toll-like receptors (TLR), expressed by the cells of the immune system, are a class of pathogen- or microbe-associated pattern recognition receptors, which play a key role in the immune recognition and initiation of host defensive responses [12]. One such receptor in avian species is TLR21, a functional ortholog of mammalian TLR9, engaged in the recognition of viral and bacterial DNA containing unmethylated cytosine-guanosine (CpG) motifs [13]. The TLR21 agonist, synthetic CpG-oligodeoxynucleotides (CpG-ODN), is categorized into three classes, class A, B, or C, whose classification is based on their effects on peripheral blood mononuclear cells and variations in their structures [14,15]. TLR agonists have been used successfully as immunostimulatory molecules, as well as adjuvants in boosting vaccine-induced immunity in chickens [16]. For example, a class B CpG-ODN molecule administered in ovo has been shown to activate monocytes, B cells, and CD4+ T cells [17,18,19], and boost immune responses against vaccine antigens [19,20,21]. Recently, we showed that an adjuvantation of rHVT-LT with CpG-ODN (class B) can enhance rHVT-LT vaccine-induced immune responses in one-day-old broiler chickens [22]. We found that the adjuvanted vaccination resulted in significantly increased expressions of interferon (IFN)γ, interleukin (IL)-1β, and inducible nitric oxide synthase (iNOS) genes in the lung and spleen tissues, as well as an enhanced splenic macrophage, γδ T cells, and activated CD4+ T cell responses.

In the present study, we extended our previous findings [22] to evaluate the protective efficacy of class B CpG-ODN adjuvated rHVT-LT vaccine in broiler chickens challenged with ILTV. To determine the possible immune mechanisms, splenic immune gene expression and cellular responses were evaluated.

## 2. Materials and Methods

### 2.1. Animals and Experimental Design

**Figure 1:**
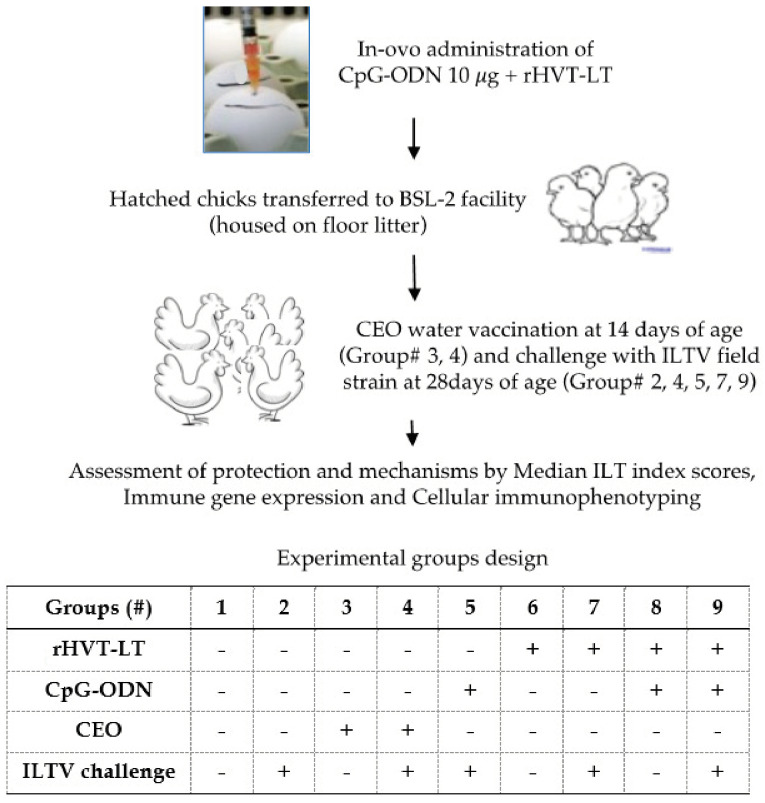
Experimental design. Schematic flow of experiments showing that embryonated eggs were in ovo vaccinated at ED18 with rHVT-LT ± CpG-ODN (10 µg per egg). Different treatment groups used in the study are tabulated at the bottom of the figure. Upon hatching, chicks were assigned individual bird numbers and housed in pens on floor litter in a BSL-2 facility. At 14 days of age, treatment groups 3 and 4 were given CEO water vaccination. Groups 2, 4, 5, 7, and 9 were challenged with ILTV intra-tracheally at 28 days of age. Clinical signs and mortality were observed for a week post-challenge followed by necropsy on day 35 of age to assess ILT index scores. Spleens were also collected at necropsy for immune gene expression using real-time PCR, and cellular immunophenotyping using flow cytometry.

All the animal experiment protocols used in this study were approved by the North Carolina State University Institutional Animal Care and Use Committee (IACUC, protocol #21-203-A). The experimental design and the treatment groups are depicted in Figure 1. Fertile Ross 708 broiler breeder chicken eggs (obtained from Aviagen Inc. Huntsville, AL, USA) were incubated and used for this experiment. A total of 375 embryonated eggs were inoculated at 18 days of embryonation, via the intra amniotic route, with either CpG-ODN (45 eggs), rHVT-LT (70 eggs), rHVT-LT + CpG-ODN (70 eggs), or just vaccine diluent (140 eggs), and the hatchability in each of these inoculations were 86.7%, 88.7%, 84.7%, and 87.5%, respectively. After hatching, the chickens were assigned to 9 treatment groups (35 chicks per group) that consisted of (1) Negative control, (2) ILTV-challenge-only control, (3) CEO-vaccine-only, (4) CEO + ILTV, (5) CpG-ODN + ILTV, (6) rHVT-LT-vaccine-only, (7) rHVT-LT + ILTV, (8) rHVT-LT + CpG-ODN, and (9) rHVT-LT + CpG-ODN + ILTV. The one-day-old chicks were individually identified by wing band and neck tag numbering before being placed on a floor litter in the BSL-2 facility animal rooms. The treatment groups were allocated to 5 different rooms to avoid any possible cross-contamination during the viral challenge period. At 14 days of age, treatment groups 3 and 4 were administered the CEO vaccine via drinking water. At 28 days of age, treatment groups 2, 4, 5, 7, and 9 were challenged with ILTV via intra-tracheal administration [9]. The birds were monitored for mortality and clinical signs (CS) post-challenge and necropsied at 35 days of age to score ILT lesions and to collect spleen samples.

An ILT index scoring system was used to quantify the severity of disease in the chickens, as previously described [9]. Briefly, the ILT index is a combination of the clinical signs (CS) (scored from 0 to 6) and the gross lesions (GL) present in the trachea at termination (score from 0 to 4) and the clinical signs (CS) (scored from 0 to 2). In order to evaluate the CS, the birds were individually observed daily from days 28 to 34 to record any clinical signs of ILTV. The CS included gasping, coughing, sneezing, conjunctivitis, and expectoration, as well as mortality. The CS calculated the number of days that a chicken was showing clinical signs. Tracheal GLs were classified in an objective rank from 0 to 4, being normal (0), presence of light mucus (1), congested, thick mucus or bloody mucus (2), presence of caseous exudate (3), and the presence of a caseous plug (4).

### 2.2. Vaccines, Adjuvant, and Virus

Two commercial vaccines were used; the CEO was obtained from Merial select, Inc. (Gainesville, GA, USA) and the rHVT-LT vaccine expressing the ILTV glycoprotein B (gB) and UL-32 proteins was obtained from Ceva Animal Health Inc. (Lenexa, KS, USA). Both the vaccines were administered following the manufacturer’s instructions. The rHVT-LT vaccine viral titration was conducted using a plaque assay, as reported previously [23]. Secondary chicken embryo fibroblasts (CEFs) were seeded with Leibovitz/McCoy medium modified with glutamine media and 4% calf serum for 24 h until a confluent monolayer formed. The monolayer was supplemented with 2% calf serum, changed every other day for the duration of the titration, and the cells were incubated at 37 °C with 5% CO_2_. Vaccine ± CpG-ODN (10 µg) were titrated in triplicate and the growth of the virus was determined through assessing the cytopathic effect on the CEFs using an inverted microscope. Titration was evaluated by counting the number of plaque-forming units (PFUs). The titer for the rHVT-LT vaccine was 2833 PFU per dose, while the CEO vaccine was used as per the manufacturer’s recommendation.

The synthetic CpG-ODN (ODN 2007, InvivoGen, San Diego, CA, USA) used in this study was a class B CpG ODN containing a full phosphorothioate backbone with one or more CpG dinucleotides. The lyophilized powder of CpG-ODN was dissolved in endotoxin-free water and stock solutions of 10 mg/mL were prepared and administered in ovo at 10 µg per egg. The CpG-ODN was mixed with the rHVT-LT vaccine in a volume of 100 µL/egg for the in ovo administrations.

The Illinois-N71851 virulent strain of ILTV was used for the challenge [9]. Birds in the challenge groups were administered ILTV (4000 PFU/bird) at 28 days of age via intratracheal inoculation.

For quantitating the rHVT-LT viral replication, splenic DNA (*n* = 6) from the broiler chickens at 35 days of age was extracted using the Puregene^®^ Tissue Core Kit (QIAGEN GmbH, Hilden, Germany). Real-time qPCR was performed to detect the transcription of a 62bp non-coding region located between the HVT (Serotype 3) genome’s ORFs, HVT072 and HVT073, along with the internal reference gene, CKN (*Gallus gallus* breed Huxu chromosome 4), using the primers given in Table 1 [8]. Gene amplification was carried out in duplicate for each sample in a 20 μL reaction using SYBR green-based master mix (PowerUp™ SYBR™, ThermoFisher Scientific Baltics UAB, Vilnius, Lithuania), and the conditions included 1 cycle of 95 °C for 10 m; 50 cycles of 95 °C for 15 s; and 60 °C for 1 m for the HVT gene, while, for the CKN gene, these were 1 cycle of 95 °C for 1 m, 55 °C for 30 s, and 95 °C for 30 s. The melting curves were created by chilling the sample at 1.6 °C/s to 55 °C and then raising the temperature by 0.1 °C/s to 95 °C at the end of the amplification.

### 2.3. Immune Gene Expression

Spleens were collected from all the treatment groups (*n =* 6) in RNAlater solution (Invitrogen, Carlsbad, CA, USA) from the chickens during terminal necropsy and stored at −80 °C until processing. The tissues were homogenized and the total RNA was extracted followed by cDNA synthesis, as described previously [22]. Quantitative real-time reverse-transcriptase PCR using SYBR Green was performed on the diluted cDNA using a QuantStudio 6 Flex System and QuantStudio Real-Time PCR Software (Applied Biosystems, Waltham, MA, USA). Each reaction involved a pre-incubation period of 50 °C for two minutes, followed by 95 °C for two minutes, followed by 35–45 cycles of 95 °C for 10 s and 55–64 °C for 5 s, depending on the primer’s binding suitability, and the elongation step was 72 °C for 10 s. A subsequent melt-curve analysis was performed by heating to 95 °C for 15 s, cooling to 60 °C for 1 min, and heating to 95 °C for 15 s. The primers for the amplification of the IFNγ, IFNβ, IL-1β, IL-12, TLR21, and iNOS genes were synthesized by Integrated DNA Technologies (Coralville, IA, USA), and the primer sequences along with the annealing temperature and gene accession number are given in Table 1. The expression levels of the target genes were calculated relative to the stably expressed reference gene, β-actin [24], using the relative gene expression method [25,26].

### 2.4. Flow Cytometry

Single-cell splenocyte preparations (*n* = 8 per group) were made following a protocol described previously [22]. The cells were stained in three different panels of antibody staining due to the paucity of chicken antibody reagents available in multi-color formats. All the anti-chicken antibodies were purchased from Southern Biotech Inc., Birmingham, AL, USA, and were of mouse origin, and their respective clones are given the parenthesis below. The first panel used antibodies against mannose receptor C1-like B (MRC1L-B) (monocyte/macrophage lineage, clone KUL01), γδTCR (TCR-1), Bu-1 (AV-20), and IgM (M1), the second panel used anti-CD3 (CT-3), CD4 (CT-4), CD28 (AV7), and CD44 (AV6), and the third panel used anti-CD3 (CT-3), CD8 (CT-8 recognizing CD8α chain), CD28 (AV7), and CD44 (AV6). In all three panels, cell viability dye, Live/Dead Near IR (Invitrogen, Calsbad, CA, USA), was used to exclude dead cells. The chicken cell markers and their corresponding antibody fluorochrome formats are given in Table 2. The stained cells along with the single-stain compensation and fluorescent minus one control were washed and fixed with 4% paraformaldehyde before data acquisition using an LSR-II flow cytometer (BD Biosciences, Franklin Lakes, NJ, USA). The data analysis was carried out using the FlowJo software v10 (Tree Starr, Ashland, OR, USA). The gating strategy included the exclusion of doublet cells through forward and side scatter plotting using the area (A), height (H), and width (W), followed by gating on live cells. Briefly, an analysis of all the samples was performed on the basic ‘live cell’ gate and the cells expressing mannose receptor C1-like B (MRC1L-B) (monocyte/macrophage lineage, clone KUL01) were gated as macrophages, TCRγδ+ cells were gated as γδ T cells, and cells expressing both Bu-1 and IgM were gated as B cells. To determine the frequencies of T cell subsets, CD4+, and CD8+, the live CD3+ population was used as the backbone gate. To evaluate T cell activation, the live CD3+ cell (T cell) population was used as the backbone gate, followed by gating on the CD4+ and CD8+ T cells expressing CD28 or CD44 costimulatory/activation molecules.

### 2.5. Statistical Analysis

All the data were analyzed using GraphPad Prism v10 (GraphPad software, San Diego, CA, USA). The data were first tested for normal distribution (Shapiro–Wilk test) followed by one-way ANOVA (parametric or non-parametric) test analyses. The ILT lesions score data were analyzed using the Kruskal–Wallis test followed by Dunn’s multiple comparison test, while the immune gene expression and the immunophenotyping data were analyzed, depending on the data distribution, using the parametric Tukey’s multiple comparisons test or the nonparametric Kruskal–Wallis ANOVA tests.

## 3. Results

### 3.1. Protection against ILTV Challenge

**Figure 2:**
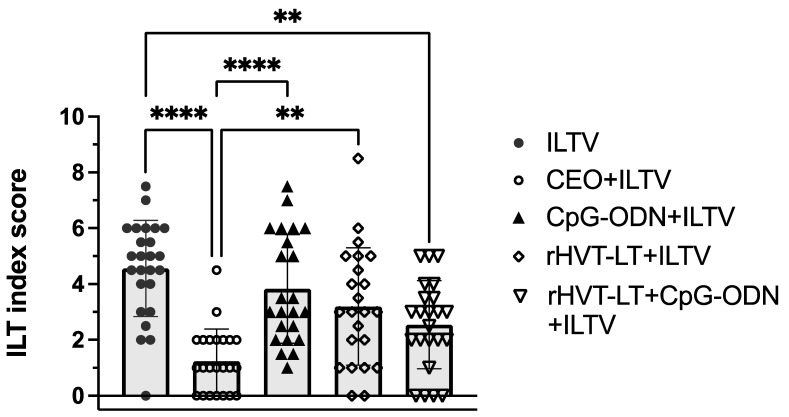
Assessment of vaccine-induced protection in chickens. Broiler chickens receiving in ovo treatments with CpG-ODN alone, rHVT-LT alone, or rHVT-LT + CpG-ODN, as well as a CEO vaccination (in water at 14 days of age), were challenged intratracheally with wild-type ILTV at 28 days of age. The clinical signs (CS) were observed for a week post-challenge followed by necropsy on day 35 of age to assess ILT lesions and determine the intratracheal pathogenicity index (ITPI). The ILT index score depicted in the figure is a combination of ITPI (scored from 0 to 4) and the CS (scored from 0 to 2). Asterisks above the group’s average plotted in the graph indicate statistical significance between the groups; ** *p* < 0.01, **** *p* < 0.0001.

To evaluate the protective efficacy of the different treatment groups against the viral challenge, the clinical severity of disease was measured in terms of ILT index score. As shown in Figure 2, a reduction (*p* < 0.05) in the ILT severity was observed in the birds administered with the rHVT-LT + CpG-ODN or CEO vaccine, when compared to the group receiving the ILTV challenge alone, indicating vaccine-induced protection in these two vaccinated groups. Additionally, the clinical severity in the CEO-vaccinated birds was lesser (*p* < 0.05) than that in the groups that received CpG-ODN or rHVT-LT only. However, no statistical difference was observed in the ILT index scores between the CEO- and rHVT + CpG-ODN-vaccinated groups. No significant protection was observed in the birds receiving CpG-ODN or rHVT-LT alone, compared to the group receiving the ILTV challenge alone.

Additionally, the rHVT viral replication in the spleens collected at necropsy from the groups receiving the rHVT-LT or rHVT-LT + CpG-ODN vaccine was determined. The results showed that, while none of the unvaccinated control birds were positive for the virus, all the chickens tested in the rHVT-LT- or rHVT-LT+ CpG-ODN-vaccinated groups were positive for rHVT, indicating vaccine viral replication in these birds.

### 3.2. Immune Gene Expression

**Figure 3:**
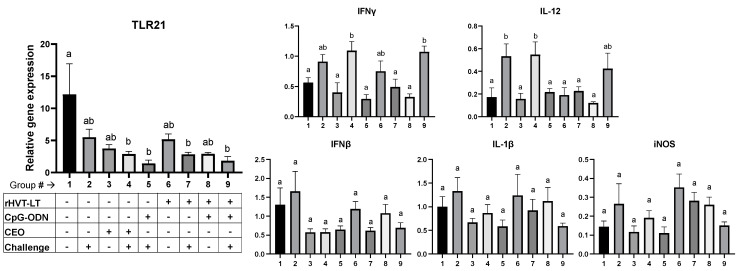
Effect of rHVT-LT, CpG-ODN adjuvantation, CEO, and ILTV on the immune gene expressions in spleen. Broiler chickens were vaccinated in ovo with rHVT-LT with or without CpG-ODN along with unvaccinated controls. A CEO vaccine was administered via drinking water at 14 days of age. Chickens were challenged intratracheally with wild-type ILTV at 28 days of age. Different treatment groups used in the study are tabulated, as shown in the figure. Necropsy was performed at 35 days of age and the spleens were collected for RNA extraction and cDNA synthesis. Real-time PCR to quantify immune gene expression along with the reference gene (β-actin) and the target gene expression levels relative to β-actin were calculated. Different letters above the standard-error-of-mean bars indicate significant difference (*p* < 0.05) between the groups.

To evaluate the effect of different treatments on the splenic immune response, the expressions of the IFNγ, IFNβ, IL-1β, IL-12, TLR21, and iNOS genes were quantified. As shown in Figure 3, a decrease (*p* < 0.05) in the expression of the TLR21 gene was observed in the ILTV-challenged groups receiving CEO (Group 4), CpG-ODN + ILTV challenge (Group 5), rHVT-LT (Group 7), or rHVT-LT + CpG-ODN (Group 9) in comparison to the negative control (untreated and unchallenged; Group 1). An upregulation (*p* < 0.05) in the gene expression of IFNγ was observed in the challenged groups receiving CEO (Group 4) or the rHVT-LT + CpG-ODN vaccine (Group 9) when compared to the negative control (Group 1), CEO (Group 3), CpG-ODN + ILTV (Group 5), rHVT-LT + ILTV (Group 7), and rHVT-LT + CpG-ODN (Group 8) groups. Additionally, the birds administered ILTV alone (Group 2) or CEO + ILTV (Group 4) had an upregulated (*p* < 0.05) transcription of IL-12 in comparison to rest of the treatments (Groups 1, 3, 5, 6, 7, and 8), except those receiving rHVT-LT + CpG-ODN + ILTV (Group 9). No significant changes were observed in the gene expressions of IFNß, IL-1ß, or iNOS between any of the treatment groups.

### 3.3. Immunophenotyping

In order to further determine the effect of the treatment groups on the splenic cellular response, the changes in the frequency of macrophages, B cells, and T cells, including their activation statuses, were evaluated.

#### 3.3.1. Macrophage, B Cell, and γδ T Cell Responses

**Figure 4:**
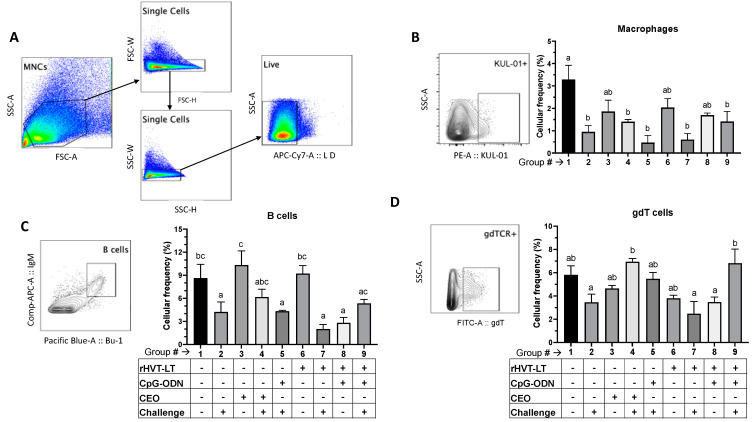
Treatment-related effects on the splenic macrophage, B cell, and γδ T cell responses. Vaccination of broiler chickens was performed in different treatment groups, as tabulated in the figure. While the rHVT-LT vaccination with or without CpG-ODN was administered in ovo, the CEO water vaccination was given at 14 days of age. Birds were challenged intratracheally with wild-type ILTV at 28 days of age and, at necropsy (35 days of age), the spleens were collected for preparing single-cell suspensions. Cells were stained with antibodies against chicken KUL-01 (macrophages), Bu-1 and IgM (B cell markers), and TCRγδ (γδ T cell) molecules for flow-cytometry-assisted immunophenotyping analysis. (**A**) Indicates the gating strategy used to exclude doublet and dead cells. Live cells were then gated to obtain populations of macrophages (**B**), B cells (**C**), and γδ T cells (**D**), and the bar graphs plot the average cellular frequency of each of the cell populations. Different letters above the stand error of means bars indicate significant difference (*p* < 0.05) between the groups.

Figure 4A depicts the gating strategy used for measuring the frequencies of the macrophages, B cells, and γδ T cells. As shown in Figure 4B, the frequency of macrophages was found to be decreased (*p* < 0.05) in the groups that received the ILTV challenge, namely Groups 2 (no treatment), 4, 5 (CpG-ODN), 7 (rHVT), and 9 (rHVT + CpG-ODN), when compared to the Group 1 untreated-unchallenged (negative control) chickens. The B cell analysis showed that the birds receiving ILTV only (Group 2), CpG-ODN + ILTV (Group 5), rHVT-LT + ILT (Group 7), and rHVT-LT + CpG-ODN (Group 8) had a reduced (*p* < 0.05) frequency of B cells (Bu-1+ IgM+) compared to the negative control (Group 1), CEO (Group 3), and rHVT-LT (Group 6) (Figure 4C) groups. No significant difference in B cell frequencies was observed between the negative control (Group 1), CEO (Group3), CEO + ILTV (Group 4), rHVT-LT (Group 6), and rHVT-LT + CpG-ODN + ILTV (Group 9) groups. Furthermore, as a shown in Figure 4D, the CEO + ILTV (Group 4) and rHVT-LT + CpG-ODN + ILTV (Group 9) groups had higher frequencies (*p* < 0.05) of γδ T cells than those in the ILTV-only (Group 2), rHVT-LT + ILTV (Group 7), and rHVT-LT + CpG-ODN (Group 8) groups; however, the increased frequencies in groups 4 and 9 were statistically similar to the negative control (Group 1). No significant difference in γδ T cells was observed between the CEO (Group 3), CpG-ODN + ILTV (Group 5), and rHVT-LT only (Group 6) groups.

#### 3.3.2. T Cell Responses

**Figure 5:**
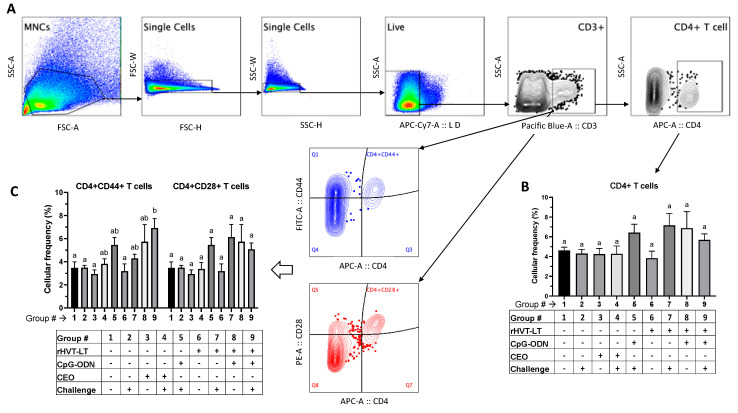
Treatment-related effects on the splenic CD4+ T cell responses. The activation of splenic CD4+ T cells was measured in broiler chickens receiving different treatments, as tabulated in the figure. Following an intratracheal challenge with wild-type ILTV at 28 days of age, the chickens were necropsied (35 days of age) to collect spleens and prepare single-cell suspensions for flow cytometry. Cells were stained with antibodies against chicken CD3, CD4, CD44, and CD28 molecules. (**A**) Indicates the gating strategy used to exclude doublet and dead cells. Live cells were then gated on CD3+ cells followed by CD4+ cells to obtain populations of CD4+ T cells (**B**). The cellular activation was measured based on the frequencies of CD4 + CD44+ and CD4 + CD28+ T cells (**C**). The bar graphs show the average cellular frequency of each of the cell populations. Different letters above the stand error of means bars indicate significant difference (*p* < 0.05) between the groups.

Figure 5A depicts the gating strategy used for evaluating the frequencies of CD4+ T cells, as well those expressing CD44 and CD28 activation molecules. While there was no significant difference in the CD4+ T cell frequency between the treatment groups (Figure 5B), the T cell activation analysis revealed the following treatment-related changes. There was a significant increase (*p* < 0.05) in the frequency of CD4 + CD44+ T cells in the chickens receiving rHVT-LT + CpG-ODN + ILTV (Group 9) when compared to the negative control (Group 1) and those administered ILTV only (Group 2), CEO (Group 3), or rHVT-LT (Group 6) (Figure 5C). However, there was no significant difference in the frequencies of these CD4 + CD44+ T cells amongst the rHVT-LT + CpG-ODN (Group 9), CEO + ILTV (Group 4), CpG-ODN + ILTV (Group 5), rHVT-LT + CpG-ODN (Group 7), and rHVT-LT + CpG-ODN (Group 8) groups. Furthermore, no significant changes were observed in the frequency of CD4 + CD28+ T cells between any of the treatment groups.

**Figure 6:**
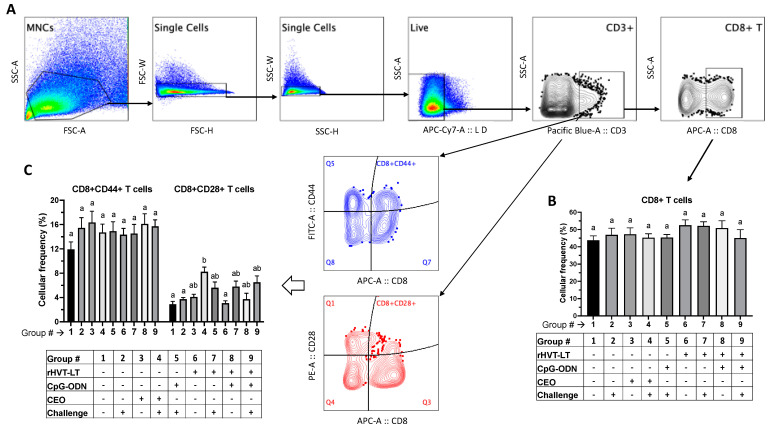
Treatment-related effects on the splenic CD8+ T cell responses. Broiler chickens receiving different treatments were challenged intratracheally with wild-type ILTV at 28 days of age. At necropsy (35 days of age), the spleens were collected and single-cell suspensions were prepared. Cells were stained with antibodies against chicken CD3, CD8, CD44, and CD28 molecules for immunophenotyping analysis. (**A**) Indicates the gating strategy used to exclude doublet and dead cells. Live cells were then gated on CD3+ cells followed by CD8+ cells to obtain populations of CD8+ T cells (**B**). The cellular activation was measured based on the frequencies of CD8 + CD44+ and CD8 + CD28+ T cells (**C**). The bar graphs show the average cellular frequency of each cell population. Different letters above the stand error of means bars indicate significant difference (*p* < 0.05) between the groups.

Figure 6A depicts the gating strategy used for measuring the frequencies of CD8+ T cells, as well those expressing CD44 and CD28 activation molecules. The frequency of CD8 + CD28+ T cells was significantly increased (*p* < 0.05) in the birds given CEO + ILTV (Group 4) compared to the negative control (Group 1), ILTV-only (Group 2), and rHVT-LT (Group 6) groups. However, there was no significant difference in the frequencies of these CD8 + CD28+ T cells between the CEO + ILTV (Group 4), CEO (Group 3), CpG-ODN + ILTV (Group 5), rHVT-LT + ILTV (Group 7), rHVT-LT + CpG-ODN (Group 8), and rHVT + CpG-ODN + ILTV (Group 9) groups. Furthermore, no significant changes were observed in the frequency of CD8+ T cells (Figure 6B) and frequency of CD8 + CD44+ T cells (Figure 6C) between any of the treatment groups.

In addition to the figures presented here, a summary highlighting the significant (*p* < 0.05) changes in the splenic cellular frequencies in the chickens challenged with or without ILTV compared to the untreated-unchallenged chickens (negative control, Group# 1) is given in Supplementary Table S1.

## 4. Discussion

In recent years, incidences of ILT, primarily in broiler flocks, have been on the rise, leading to significant economic losses [5]. Although the live-attenuated CEO vaccine can effectively prevent the clinical disease and its mortalities, the major drawback is that the CEO vaccine strain can regain virulence through bird-to-bird passages, thus inducing mortality and severe respiratory reactions [2,3,4]. While the rHVT-LT vaccine can be advantageous in that it can reduce clinical signs and is approved for in ovo delivery, its failure to protect chickens against ILT as effectively as the CEO vaccine warrants the need for more efficacious recombinant vaccines. Recently, we reported that an in ovo adjuvantation of the rHVT-LT vaccine with CpG-ODN can enhance vaccine-induced immune responses in one-day-old broiler chickens [22]. In the present study, we evaluated the protective efficacy of the CpG-ODN-adjuvated rHVT-LT vaccine in broiler chickens. The findings revealed that 1) in ovo rHVT-LT + CpG-ODN vaccination can provide anti-ILT protection to a level similar to that of CEO, and 2) both the CpG-ODN + rHVT-LT and CEO vaccinations can enhance the T helper-1 (Th1) cytokine response, along with increased γδ T and activated CD4+ or CD8+ T cell responses.

Vaccine-induced protection against ILT is often measured by evaluating the mortality and the severity of the clinical disease, including the clinical signs and lesions in the trachea of affected birds [2]. In the present study, CpG-ODN-adjuvated rHVT-LT vaccination provided significant protection against ILTV challenge. Importantly, the levels of protection induced by the rHVT-LT + CpG-ODN and the CEO vaccines were statistically similar, suggesting that CpG-ODN can be an effective adjuvant for rILT vaccines. Our previous work showed that CpG-ODN possesses immunostimulatory properties and can promote antiviral effects in chickens [17,18,20,22,27,28]. Although the rHVT-LT vaccination alone did not result in statistically significant protection compared to the control, a numerical decrease in the ILT indices was observed in the vaccinated group. This observation supports the previous findings, indicating that the level of protection conferred by rHVT-LT against a challenge with virulent ILTV has been somewhat contentious [9,29]. It was noteworthy that CEO-induced protection was found to be more robust than that of the rHVT-LT + CpG-ODN vaccination, since the ILT index score in the CEO group was significantly lower than that given either by CpG-ODN or rHVT-LT alone; such a protective ability of the CEO vaccine against ILT in an experimental setting has also been previously noted [10,11,30,31]. However, in commercial broiler operations, the CEO vaccine is often proved to be ineffective due its poor flock vaccination coverage, inadequate biosecurity measures, and the chances of concomitant respiratory infections [2]. Additionally, we and others have shown that CEO vaccines can contribute to recombination with other vaccine(s) or wild-type ILTV, resulting in CEO virus revertants with an enhanced virulence ability in establishing a latency in apparently healthy chickens [7,32]. In this context, the present study findings suggested that adjuvanted rILT vaccines that are safe, approved for in ovo administration, and devoid of virus-regaining virulence issues can be worth consideration for commercial application in inducing anti-ILT protective immunity.

Immune protection against ILTV is often mediated by the Th1-response, orchestrated by activated CD4+ and CD8+ T cells, whose functions are chiefly driven by cytokines such as IFNγ and IL-12 [33]. The present study found that the splenic IFNγ transcription in the viral-challenged chickens vaccinated with the rHVT-LT + CpG-ODN or CEO vaccines was significantly higher than that in the untreated-unchallenged (negative control) birds. Importantly, the rHVT-LT + CpG-ODN-vaccinated birds had a significantly higher IFNγ gene expression compared to those receiving rHVT-LT without CpG-ODN, indicating the adjuvant-induced augmentation of this cytokine. Previous studies have shown that IFNγ plays a pivotal role in anti-ILTV defense in chickens [33,34], and that the in ovo administration of CpG-DNA can augment IFNγ induction in ILTV, avian influenza virus, or infectious bronchitis virus-challenged chicks [17,18,29]. More recently, we found that the in ovo administration of rHVT-LT + CpG-ODN can induce increased transcriptions of IFNγ and IL-12 in the spleen of one-day-old broiler chicks [22]. Although we did not observe any significant changes in the splenic IL-12 transcription in the birds receiving rHVT-LT + CpG-ODN compared to the control in the present study, the CEO-vaccinated group had a significantly higher expression of this cytokine, suggesting a more robust Th1-driven protective response. Furthermore, when compared to the ILTV-challenge-only control group, the birds in the CEO-vaccinated group had a significantly higher splenic frequency of CD8 + CD28+ T cells, while the frequency of CD4 + CD44+ T cells was significantly increased in the rHVT-LT + CpG-ODN-vaccinated group, suggesting the role of activated T cells in the Th1-mediated protective immunity of these two groups [10,35,36]. It is noteworthy that, while CD28 is a costimulatory receptor expressed on activated T cells [37], CD44 is an adhesion receptor involved in activated T cell migration between lymphoid tissues and sites of infection [38]. The key roles of CD4+ and CD8+ T cells in protection against avian viruses, including ILTV, have previously been reported [33,35,39]. Additionally, our previous work investigating the role of CpG-ODN in the host defense against ILTV and other viruses indicated immunostimulatory effects of this molecule in augmenting the CD4+ T cell response in chicks [18,29]. The subtle difference in the splenic activation of CD4+ versus CD8+ T cell-type between the rHVT-LT + CpG-ODN and CEO vaccinations suggested that either CpG-ODN favors the activation of CD4+ T cells over CD8+ T cells [40], or the observed effects in both the vaccine groups were tissue-specific [18], which needs further investigation. It is also noteworthy here that the CT-8 antibody clone used for staining the CD8^+^ cells recognizes the α-chain. Hence, our evaluation of the CD8^+^ T cells included both CD8αα^+^ and CD8αβ^+^ cells; the latter are presumably considered to function as cytotoxic T lymphocytes [39], which may have contributed to more robust CEO-induced protective immunity [36]. Although the Th1 response is known to play a key role in antiviral immunity, it can also be a limitation if the expression of pro-inflammatory mediators is not regulated. Such modulation of the expression of pro-inflammatory mediators in lymphoid organs following an ILTV infection occurs via the expression of anti-inflammatory cytokines such as IL-10 [41]. Although we did not evaluate IL-10 expression in our experiment, there was some evidence observed in relation to the regulation of proinflammatory mediators, namely IL-1β and iNOS in the CEO-vaccinated and rHVT-LT + CpG-ODN-vaccinated groups. However, more work involving the evaluation of IL-10 or IL-13 gene expression, as well as macrophage and T cell analyses in other tissues (trachea, lungs, and Harderian glands), seems to provide a more in-depth mechanistic viewpoint.

The present study also investigated changes in the splenic frequencies of γδ T cells and macrophages, which are important in the avian host innate antiviral defense. Upon encountering the pathogen, γδ T cells can rapidly secrete effector cytokines and exert cytotoxicity [42], while macrophages respond via phagocytosis and cytokine production [43]. The frequencies of γδ T cells in the ILTV-challenged groups receiving the rHVT-LT + CpG-ODN or CEO vaccines were significantly higher than those receiving the challenge only or rHVT-LT only, suggesting an important role for these cells in protection against ILT. In support of this observation, our recent study had found that in the ovo administration of the CpG-ODN-adjuvanted rHVT-LT vaccine can induce an increased proliferation of splenic γδ T cells in one-day-old chickens [22]. Previous studies have also indicated a protective role of γδ T cells in the antiviral defense of chickens [44]. In the context of macrophages, the present study found that all the ILTV-challenged groups had a significantly reduced splenic frequency of KUL-01+ cells when compared to the negative control, indicating a likely recruitment of these splenic macrophages to the respiratory sites of infection in response to ILTV. This is because we previously observed that an infection of chickens with wild-type (AB-S63) and CEO vaccine revertant (AB-S45) ILTV strains led to a significantly higher recruitment of macrophages to the trachea and lungs at 3 and 7 days post-infection [45]. Additionally, we also reported earlier that CpG-ODN alone or in combination with rHVT-LT administration in ovo results in a significant increase in the splenic population of KUL-01+ macrophages at 1 or 3 days post-hatch [18,22], thus supporting the present study’s notion of macrophage recruitment from the spleen to the respiratory tissues following a viral challenge. An intriguing finding in the present study was also that the splenic TLR21 gene expression was found to be significantly decreased in the ILTV-challenged chickens receiving CEO, CpG-ODN only, rHVT-LT only, or rHVT-LT + CpG-ODN when compared to the unchallenged negative control group. This observation may perhaps possibly have been due to (1) a migration of TLR21-expressing macrophages away from the splenic tissues, (2) herpesvirus-mediated receptor downregulation [22,46], or (3) an altered rate of splenic TLR21 transcription [47] in the chickens. Interestingly, the splenic B cell frequencies in some of the ILTV-challenged groups, namely those receiving no treatment, CpG-ODN only, or rHVT-LT only in the present study, were also significantly lower than those in the negative control group. Further mechanistic investigation is needed to determine the precise role of B cells in anti-ILTV responses in relation to rHVT-LT or CEO vaccination.

## 5. Conclusions

In the present study, CpG-ODN-adjuvanted rHVT-LT in ovo vaccination provided significant protection against ILT in broiler chickens, to a level similar to that of the CEO vaccine. The immune protection mechanisms included a Th1-mediated immune response, characterized by an upregulated expression of the IFNγ and/or IL-12 cytokine genes, and increased frequencies of γδ T cells and activated CD4+/CD8+ T cells.

## Figures and Tables

**Table 1:** Sequences for the primers used in real-time PCR assays.

Target Gene	Primer Sequence	Annealing Temp (°C)	Accession Number
HVT-TM-2	F: 5′-CGGGCCTTACGTTTCACCT-3′ R: 5′-GCGCCGAAAAGCTAGAAAAG-3′	60	NC_002641.1
CKN	F: 5′-GCTACAGCGAGCTCATTTTTTTAGT-3′ R: 5′-TTTACAATGGGTTTAGGTGTCTGAGA-3′	55	CP100558.1
β-actin	F: 5′-CAACACAGTGCTGTCTGGTGGTA-3′ R: 5′-ATCGTACTCCTGCTTGCTGATCC-3′	58	X00182
IFNγ	F:5′-ACACTGACAAGTCAAAGCCGCACA-3′ R:5′-AGTCGTTCATCGGGAGCTTGGC-3′	60	X99774
IFNß	F:5′- CGTGTGCGAGAACAGCATGGAGA-3′ R:5′-TCAGGCATTTCTCCTCGTCGAAGC-3′	60	NM_204628.1
IL-1ß	F:5′-AGCAGATCAAGGAGACGTTC-3′ R:5′-ATCAGCAGGTACTCCTCGAT-3′	55	AJ621614
IL-12	F:5′-CCAAGACCTGGAGCACACCGAAG-3′ R:5′-CGATCCCTGGCCTGCACAGAGA-3′	64	AY262752.1
TLR21	F:5′-CCTGCGCAAGTGTCCGCTCA-3′ R:5′-GCCCCAGGTCCAGGAAGCAG-3′	60	NM_001030558.1
iNOS	F: 5′-CCTGGTGATGCTGTGAATTG-3′ R: 5′-CTTCTGTGTCGTTGCATTCAG-3′	58	NM_204665

**Table 2:** Cellular markers and the fluorescent antibody format/conjugate used in flow cytometry staining.

	Marker	Format
Panel 1	KUL-01	PE
	IgM	APC
	Bu-1	Pacific Blue
	γδTCR	FITC
	Live/Dead viable dye	APC-Cy7
Panel 2	CD3	Pacific Blue
	CD4	APC
	CD28	PE
	CD	FITC
	Live/Dead viable dye	APC-Cy7
Panel 3	CD3	Pacific Blue
	CD8	APC
	CD28	PE
	CD44	FITC
	Live/Dead viable dye	APC-Cy7

## Data Availability

Research data available upon request from R.R.K.

## Supplementary Materials

The following supporting information can be downloaded at: https://www.mdpi.com/article/10.3390/v15102103/s1, Table S1: Summary of statistically significant changes in the splenic cellular frequencies in chickens challenged with or without ILTV compared to the untreated-unchallenged chickens (negative control, Group# 1). + denotes significantly increased (*p* < 0.05); - denotes significantly decreased (*p* < 0.05); = denotes no significant changes.
