# Supplementary material for: In Ovo Vaccination with Recombinant Herpes Virus of the Turkey-Laryngotracheitis Vaccine Adjuvanted with CpG-Oligonucleotide Provides Protection against a Viral Challenge in Broiler Chickens
Source: Viruses. 2023 Oct 17;15(10):2103. doi: 10.3390/v15102103 (PMC10612038; doi:10.3390/v15102103)
Supplement: Supplementary file 1 [file viruses-15-02103-s001.zip › viruses-2657348-supplementary.pdf]

Supplementary Table S1. Summary of statistically significant changes in the splenic cellular frequencies in chickens challenged with or without ILTV compared to the untreated-unchallenged chickens (negative control, Group# 1). + denotes significantly increased ($p < 0.05$); - denotes significantly decreased ($p < 0.05$); = denotes no significant changes.

Cell-types	Gp# 2	Gp# 3	Gp# 4	Gp# 5	Gp# 6	Gp# 7	Gp# 8	Gp# 9
Macrophages	-	=	-	-	=	-	=	-
γδT Cells	=	=	=	=	=	=	=	=
B Cells	-	=	=	-	=	-	-	=
CD4+CD44+ T Cells	=	=	=	=	=	=	=	+
CD4+CD28+ T Cells	=	=	=	=	=	=	=	=
CD4+ T Cells	=	=	=	=	=	=	=	=
CD8+CD44+ T Cells	=	=	=	=	=	=	=	=
CD8+CD28+ T Cells	=	=	+	=	=	=	=	=
CD8+ T Cells	=	=	=	=	=	=	=	=

Groups (#)	1	2	3	4	5	6	7	8	9
rHVT-LT	–	–	–	–	–	+	+	+	+
CpG-ODN	–	–	–	–	+	–	–	+	+
CEO	–	–	+	+	–	–	–	–	–
ILTV challenge	–	+	–	+	+	–	+	-	+
